# Supplementary material for: Antibody affinity maturation and plasma IgA associate with clinical outcome in hospitalized COVID-19 patients
Source: Nat Commun. 2021 Feb 22;12:1221. doi: 10.1038/s41467-021-21463-2 (PMC7900119; doi:10.1038/s41467-021-21463-2)
Supplement: Supplementary file 1 — Supplementary Information [file 41467_2021_21463_MOESM1_ESM.pdf]

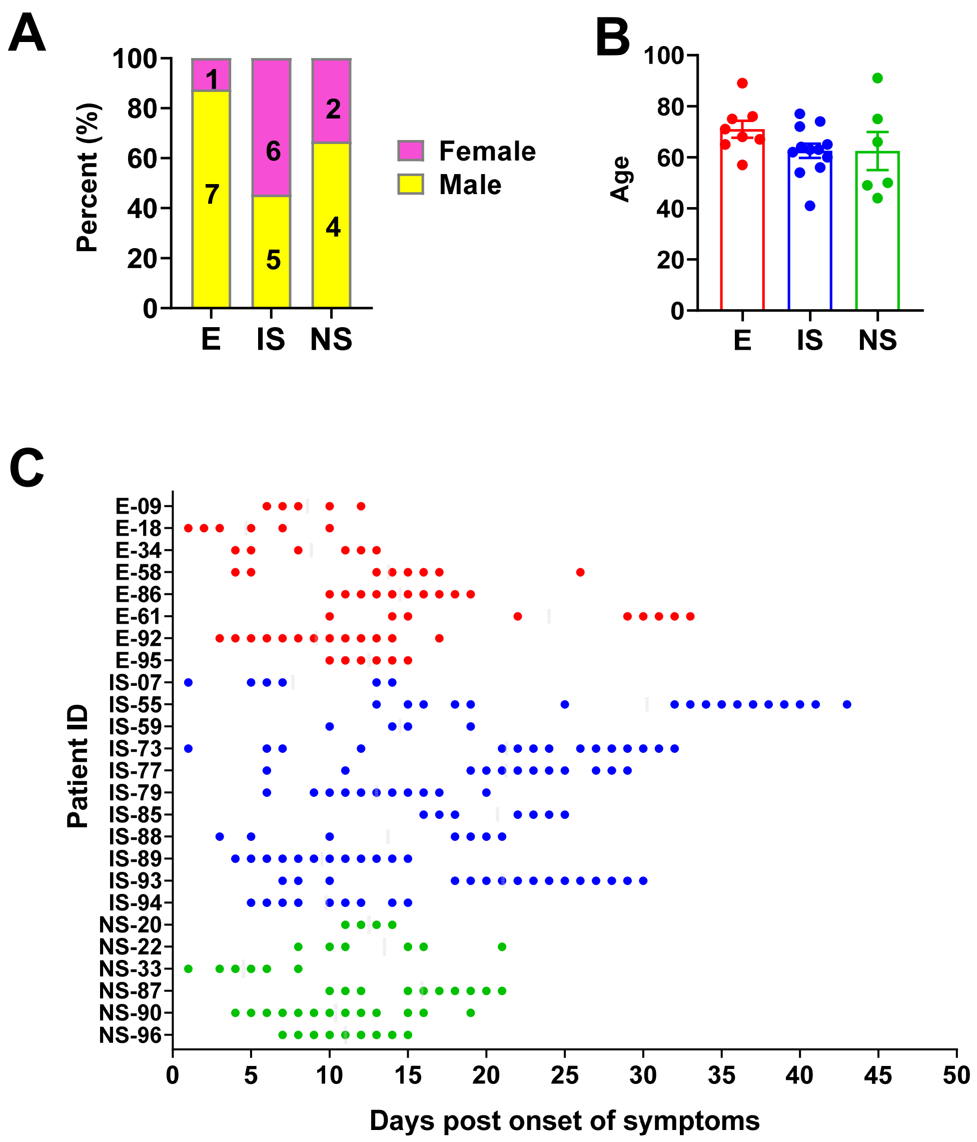

Supplementary Figure 1: Demographic characteristics and distribution of COVID-19 patients and days post onset of symptoms of plasma samples used in the study.

A) Gender distribution of patients, B) Age distribution of patients in each of the groups presented as mean values ± SEM, C) Distribution of days post onset of symptoms for each patient. E- expired patients (red; n=8 biologically independent individuals); IS- ICU admitted survived patients (blue; n=11 biologically independent individuals); NS- non-ICU survived patients (green; n=6 biologically independent individuals). The statistical significances between the groups were determined by non-parametric (Kruskal-Wallis) statistical test using Dunn's post-hoc analysis in GraphPad prism between 'expired' patients (n=8), 'ICU-survived' patients (n=11), and 'non-ICU survived' patients (n=6) did not identify any statistical significance ($p>0.05$).

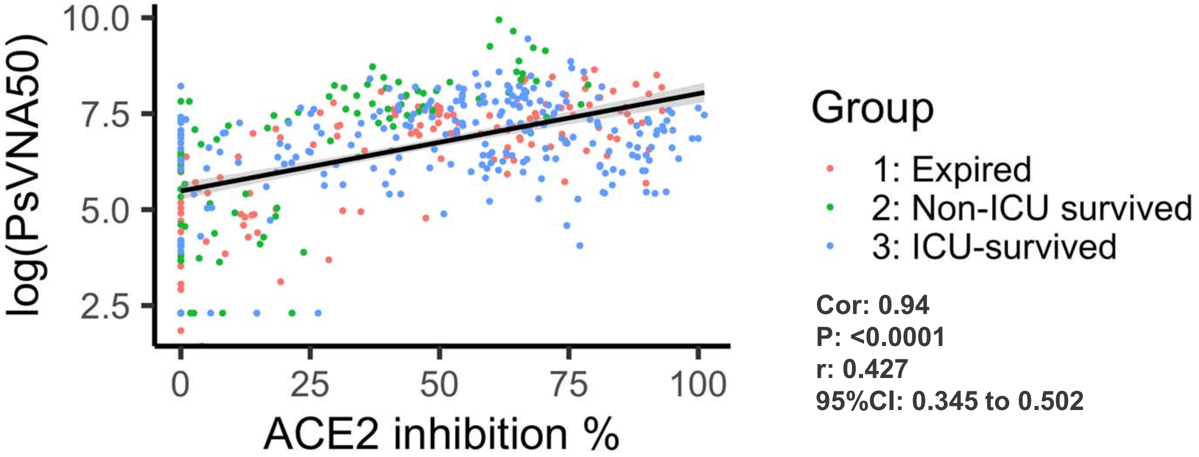

Supplementary Figure 2: Correlation of neutralization assay and ACE2 inhibition assay for measuring antibodies in the COVID-19 patients' plasma. Scatter plot of PsVNA50 (IC50 neutralization titers) and percent ACE2 inhibition of all samples in the study [Expired patients (red); ICU-survived patients (blue); non-ICU survived patients (green)]. Samples with neutralization titers of <20 were given value of 10 for graphing and calculation. Scatter plot was created with R ggplot2 package using linear regression method. The trend line is depicted as solid black line with the error bands representing 95% confidence interval. Non-parametric spearman correlation (two-sided p-value) between percent ACE2 inhibition and PsVNA50 value was calculated with Graphpad prism version 8.

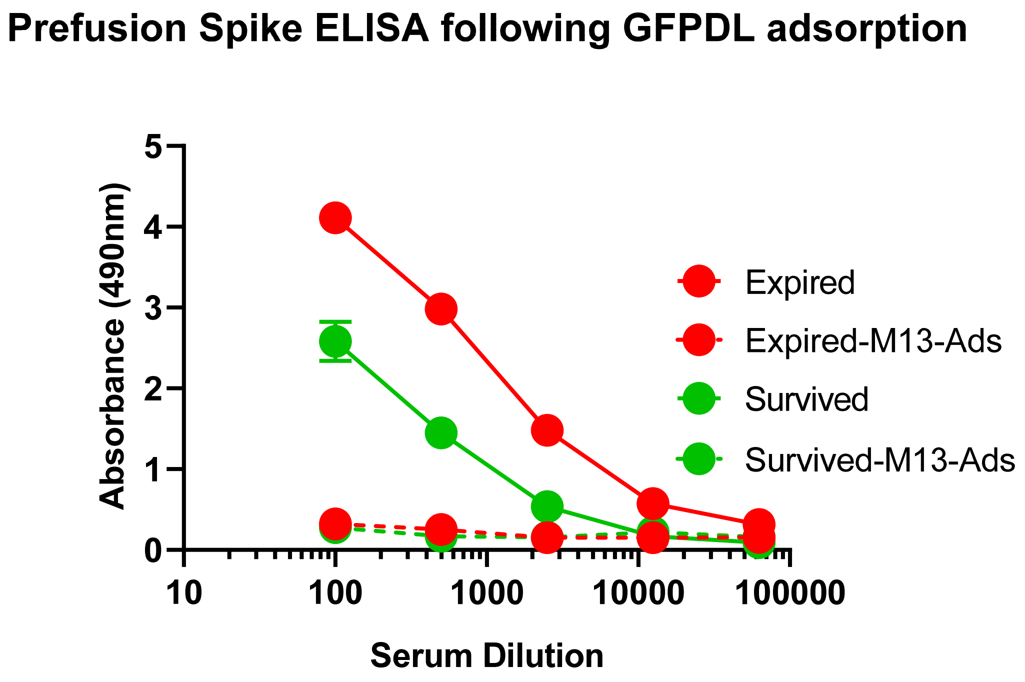

Supplementary Figure 3: Anti-prefusion SARS-CoV-2 spike reactivity of COVID-19 plasma in ELISA before and after SARS-CoV-2 GFPDL adsorption. COVID-19 sera pooled from either Expired (red; n=3) or Survivors (green; n=2) were adsorbed on SARS-CoV-2 GFPDL coated petri dishes. Binding to recombinant SARS-CoV-2 prefusion spike protein is shown before (solid lines) and after (dashed lines) GFPDL-adsorption in ELISA using HRP-conjugated goat anti-human IgA + IgG + IgM specific antibody. Line plots with error bars are presented as mean values ± SD.

Supplementary Figure 4: IgM, IgG and IgA antibody repertoires elicited in ICU-admitted surviving COVID-19 patients (IS-07, IS-88, and IS-89). Related to figure 3. (A) Distribution of phage clones after affinity selection on post-SARS-CoV-2 infection samples. Number of IgM, IgG and IgA bound phage clones selected using SARS-CoV-2 spike GFPDL on pooled polyclonal samples from days 1-4 (ICU-Survived - <D4) following symptom onset and the day of discharge (ICU-Survived - Discharged) in 'survived' ICU COVID-19 patients (IS-07, IS-88 and IS-89), and. (B-D) IgM, IgG and IgA antibody epitope repertoire recognized in the SARS-CoV-2 infected pooled plasma of 'ICU-survived' COVID-19 patients at different days post-onset of symptoms and their alignment to the spike protein of SARS-CoV-2. Graphical distribution of representative clones with a frequency of >2, obtained after affinity selection, are shown. The horizontal position and the length of the bars indicate the peptide sequence displayed on the selected phage clone to its homologous sequence in the SARS-CoV-2 spike on alignment. The thickness of each bar represents the frequency of repetitively isolated phage. Scale value for IgM, IgG and IgA is shown enclosed in a red box beneath the respective alignments. The GFPDL affinity selection data was performed in duplicate (two independent experiments by researcher in the lab, who was blinded to sample identity), and similar number of phage clones and epitope repertoire was observed in both phage display analysis.

A

	ICU SURVIVED (T0<D4)	ICU SURVIVED (DISCHARGED)
IgM	5.14E+05	2.42E+05
IgG	4.1E+04	5.6E+05
IgA	1.71E+05	7.00E+04

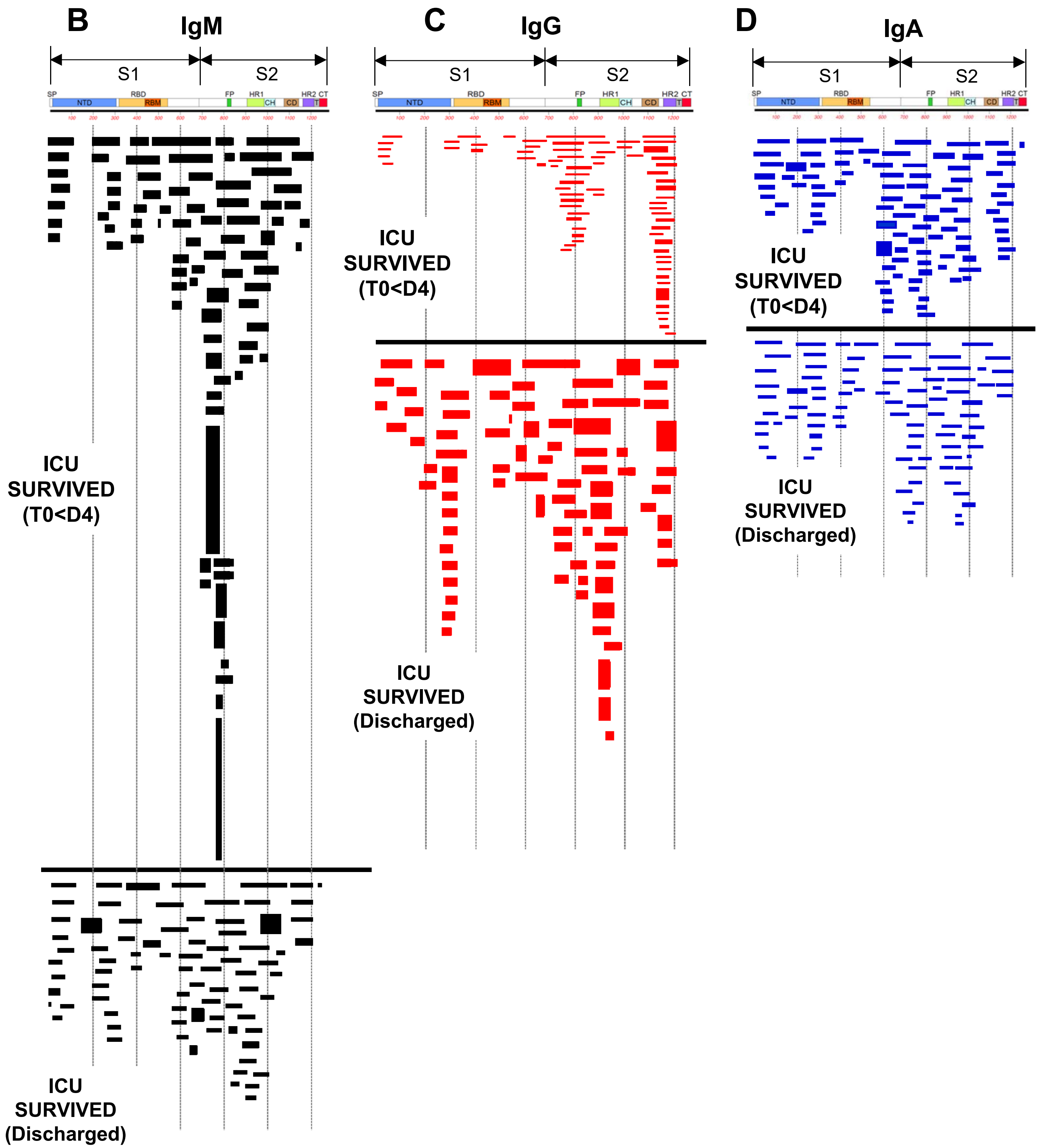

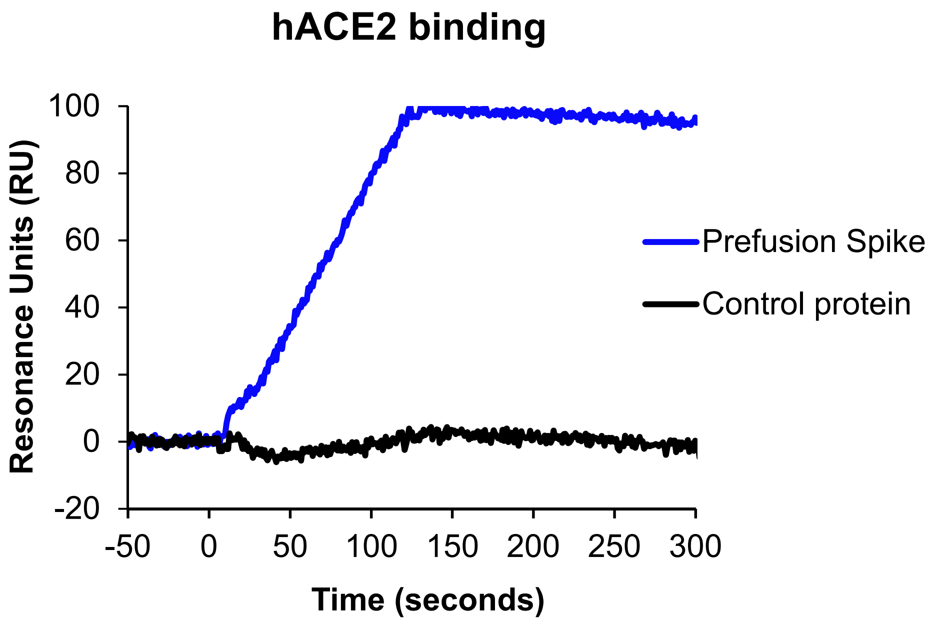

Supplementary Figure 5: **SARS-CoV-2 prefusion spike binding to hACE2 receptor in SPR. Related to figure 5 and 6.** Binding of purified prefusion spike protein to human ACE2 proteins in SPR. Sensorgrams represent binding of purified spike and control protein on low-density His-captured chips to 5 μg/mL human ACE2 protein.

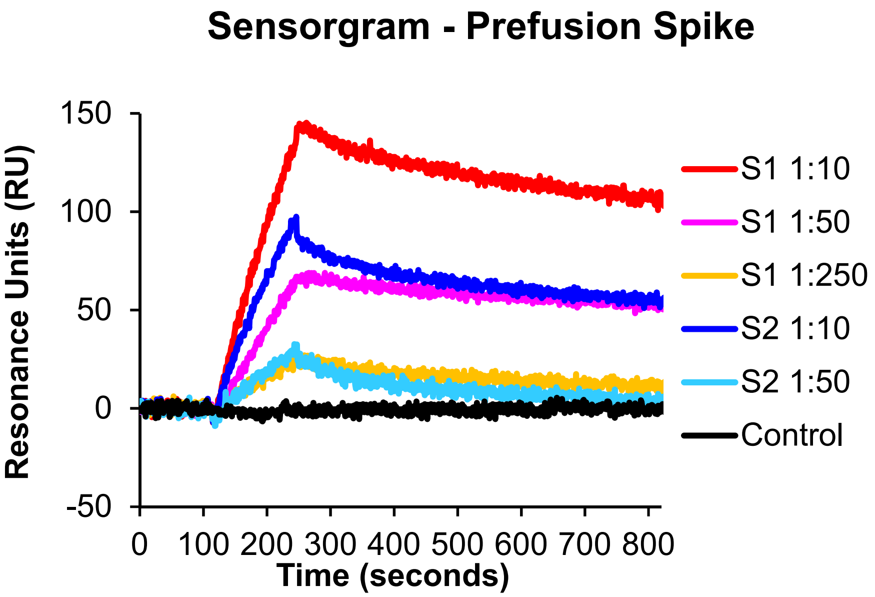

Supplementary Figure 6: Steady-state equilibrium analysis of COVID-19 antibodies binding by SPR. Related to figure 5 and 6. Serial dilutions of COVID-19 representative patient samples S1 and S2 were injected simultaneously SARS-CoV-2 prefusion spike ectodomain captured on a Ni-NTA sensor chip and on a surface free of protein (used as a blank). Binding responses from the protein surface were corrected for the response from the mock surface and for responses from a separate, buffer only injection. Uninfected control human sample at 10-fold dilution did not show any binding in SPR. Antibody off-rate constants, which describe the fraction of antigen-antibody complexes that decay per second, were determined directly from the serum sample interaction with prefusion spike using SPR in the dissociation phase only for the sensorgrams with Max RU in the range of 10-100 RU and calculated using the BioRad ProteOn manager software for the heterogeneous sample model.

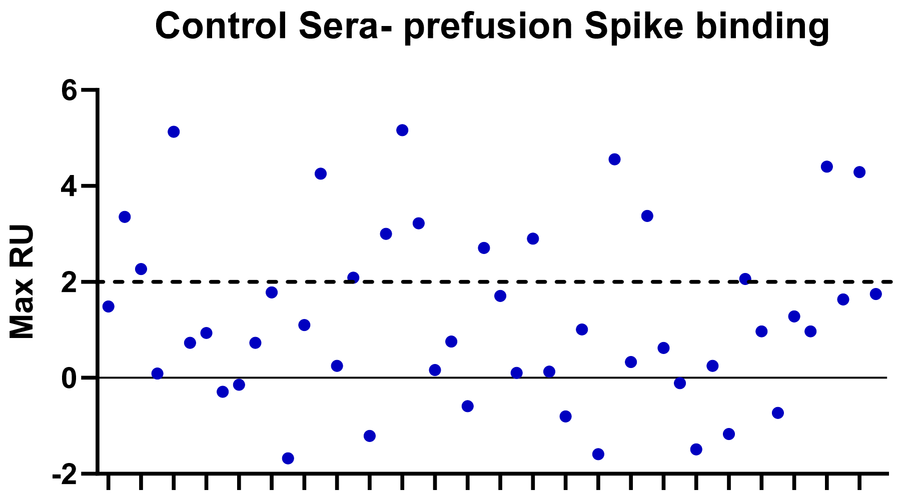

Supplementary Figure 7: Steady-state equilibrium analysis of control human samples by SPR. Related to figure 5 and 6. Ten-fold dilution of 48 control human samples collected prior to 2010 were analyzed for binding to prefusion SARS-CoV-2 spike by SPR

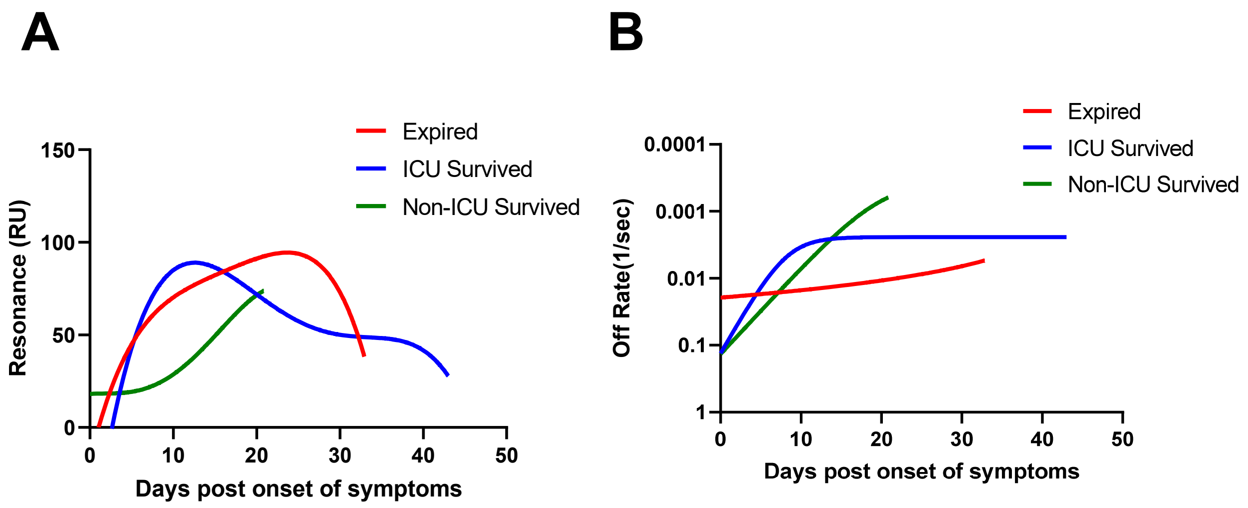

Supplementary Figure 8: Trend analysis of total prefusion spike binding antibodies and antibody affinity (off-rate) across the three groups

(A) Figure shows resonance trend lines for expired (red), ICU survivors (blue) and non-ICU survivors (green) fitted with a non-linear least squares fit of a fourth order polynomial model using Graphpad Prism (B) Antibody affinity (off-rate) trend lines for expired (red), ICU survivors (blue) and non-ICU survivors (green) fitted with a non-linear robust regression of the one phase dissociation kinetics using Graphpad Prism. Best-fit trendlines are depicted.

Supplementary Table 1: Power Analysis of Sample Size

		Expired	ICU survived	Non-ICU Survived
**Actual sample size (n)**		**8**	**11**	**6**
	**Power (1-β) & Significance (α) value**			
Calculated Sample size	1-β = 0.95, α = 0.05	5.37	5.37	5.37
	1-β = 0.9, α = 0.05	4.27	4.27	4.26
	1-β = 0.8, α = 0.05	3.08	3.09	3.09

Table shows calculated minimum N values required for an experiment to be statistically sound thereby eliminating Type I and Type II errors. Power analysis calculations were done using a 1-β value (power) as 0.95, 0.9 and 0.8 in the order of decreasing stringency, and significance (α) level of 0.05 was used. A critical Z normal table was used to look up Z_β and Z_α values in each case. The SPR determined antibody binding or antibody affinity (off-rates) to prefusion SARS-CoV-2 spike protein of each group (Expired, ICU-survived and non-ICU-survived) was calculated at around two weeks post symptom-onset (day 15) and were used for sample size calculations for each group (For e.g., off-rate of 0.0134 units was used for the Expired group and 0.00367 units was used for the ICU-survived group and 0.00974 for Non-ICU survived) as per supplementary equation 1.

Supplementary equation 1:
$$n = \left\{\frac{(|Z\alpha| + |Z\beta|)\sigma}{|\mu * - \mu o|}\right\}^2$$

where,
n- is the minimum number of samples required
σ- is the standard deviation
μ*- is the average of the study group
μ°- is the average of the control group
