# Supplementary material for: Antibody affinity maturation and plasma IgA associate with clinical outcome in hospitalized COVID-19 patients
Source: Nat Commun. 2021 Feb 22;12:1221. doi: 10.1038/s41467-021-21463-2 (PMC7900119; doi:10.1038/s41467-021-21463-2)
Supplement: Supplementary file 3 — Reporting summary [file 41467_2021_21463_MOESM3_ESM.pdf]

nature research

Corresponding author(s): Surender Khurana

Last updated by author(s): Dec 29, 2020

# Reporting Summary

Nature Research wishes to improve the reproducibility of the work that we publish. This form provides structure for consistency and transparency in reporting. For further information on Nature Research policies, see our Editorial Policies and the Editorial Policy Checklist.

## Statistics

For all statistical analyses, confirm that the following items are present in the figure legend, table legend, main text, or Methods section.

| n/a | Confirmed | |
|---|---|---|
| ☐ | ☒ | The exact sample size ($n$) for each experimental group/condition, given as a discrete number and unit of measurement |
| ☐ | ☒ | A statement on whether measurements were taken from distinct samples or whether the same sample was measured repeatedly |
| ☐ | ☒ | The statistical test(s) used AND whether they are one- or two-sided<br>*Only common tests should be described solely by name; describe more complex techniques in the Methods section.* |
| ☐ | ☒ | A description of all covariates tested |
| ☐ | ☒ | A description of any assumptions or corrections, such as tests of normality and adjustment for multiple comparisons |
| ☐ | ☒ | A full description of the statistical parameters including central tendency (e.g. means) or other basic estimates (e.g. regression coefficient) AND variation (e.g. standard deviation) or associated estimates of uncertainty (e.g. confidence intervals) |
| ☐ | ☒ | For null hypothesis testing, the test statistic (e.g. $F$, $t$, $r$) with confidence intervals, effect sizes, degrees of freedom and $P$ value noted<br>*Give P values as exact values whenever suitable.* |
| ☒ | ☐ | For Bayesian analysis, information on the choice of priors and Markov chain Monte Carlo settings |
| ☒ | ☐ | For hierarchical and complex designs, identification of the appropriate level for tests and full reporting of outcomes |
| ☐ | ☒ | Estimates of effect sizes (e.g. Cohen's $d$, Pearson's $r$), indicating how they were calculated |

*Our web collection on statistics for biologists contains articles on many of the points above.*

## Software and code

Policy information about availability of computer code

| | |
|---|---|
| Data collection | BioRad ProteON Manager software (Version 3.1.0) to collect antibody binding data from SPR machine (www.Biorad.com) |
| Data analysis | BioRad ProteON Manager software (Version 3.1.0) for antibody binding analysis from SPR machine (www.Biorad.com). Statistical analysis were performed using GraphPad prism version 8 (Graph Pad software Inc, San Diego, CA). Sequence alignments were performed using MacVector version 17.5.2 (MacVector Inc, Apex, NC). Sequence Identity was calculated using BioEdit version 7.1 (http://www.mbio.ncsu.edu/BioEdit/bioedit.html). Structures were visualized and annotated using Chimera version 1.11.2 (https://www.cgl.ucsf.edu/chimera/). |

For manuscripts utilizing custom algorithms or software that are central to the research but not yet described in published literature, software must be made available to editors and reviewers. We strongly encourage code deposition in a community repository (e.g. GitHub). See the Nature Research guidelines for submitting code & software for further information.

## Data

Policy information about availability of data

All manuscripts must include a data availability statement. This statement should provide the following information, where applicable:

- Accession codes, unique identifiers, or web links for publicly available datasets
- A list of figures that have associated raw data
- A description of any restrictions on data availability

All data are shown in the manuscript figures and supplementary information. The source data are provided in Supplementary tables 1, 3 & 4. Antigenic sites were depicted on the SARS-CoV-2 spike structure PDB#6VSB (https://www.rcsb.org/structure/6VSB). Sequence for SARS CoV-2 spike protein (Genbank#MN908947), SARS CoV-1 BJ01 strain (Genbank#AAP30030.1), MERS CoV KOR/KNIH/2015(Genbank#AKN11075.1), Bat SARS-like CoV ZC45 (Genbank#AVP78031.1), Human CoV NL63 (NCBI#YP_003767.1), and Human CoV HKU1 (NCBI#YP_173238.1) were downloaded from https://www.ncbi.nlm.nih.gov/genbank/.

# Field-specific reporting

Please select the one below that is the best fit for your research. If you are not sure, read the appropriate sections before making your selection.

☒ Life sciences  ☐ Behavioural & social sciences  ☐ Ecological, evolutionary & environmental sciences

For a reference copy of the document with all sections, see nature.com/documents/nr-reporting-summary-flat.pdf

# Life sciences study design

All studies must disclose on these points even when the disclosure is negative.

| | |
|---|---|
| Sample size | All samples from the SARS-CoV-2 infected individuals were analyzed in this study |
| Data exclusions | No data was excluded |
| Replication | GFPDL and SPR analysis were performed twice by independent researchers in the lab. The replications were successful. The variation in duplicate experimental runs was <5%. |
| Randomization | All samples from the COVID-19 patients were analyzed in this study. The study was non-randomized performed during the pandemic on hospitalized COVID-19 patients. Initially, no patient information was provided, and all the immune analyses were conducted blindly. The participants were adults and they were assigned in each experimental group based on their hospitalization status and clinical outcome. |
| Blinding | Experiments were performed by different investigators, who were blinded to sample identity. |

# Reporting for specific materials, systems and methods

We require information from authors about some types of materials, experimental systems and methods used in many studies. Here, indicate whether each material, system or method listed is relevant to your study. If you are not sure if a list item applies to your research, read the appropriate section before selecting a response.

## Materials & experimental systems

| n/a | Involved in the study | |
|---|---|---|
| ☐ | ☒ | Antibodies |
| ☐ | ☒ | Eukaryotic cell lines |
| ☒ | ☐ | Palaeontology and archaeology |
| ☒ | ☐ | Animals and other organisms |
| ☐ | ☒ | Human research participants |
| ☒ | ☐ | Clinical data |
| ☒ | ☐ | Dual use research of concern |

## Methods

| n/a | Involved in the study | |
|---|---|---|
| ☒ | ☐ | ChIP-seq |
| ☒ | ☐ | Flow cytometry |
| ☒ | ☐ | MRI-based neuroimaging |

## Antibodies

| | |
|---|---|
| Antibodies used | HRP-conjugated goat anti-human IgA + IgG + IgM (Cat No #109-035-064) and HRP-conjugated goat anti-human IgG-Fc specific antibody (Cat no #709-005-098) were purchased from Jackson Immuno Research. |
| Validation | These are secondary antibodies, which were characterized by the manufacturer (https://www.jacksonimmuno.com/) |

## Eukaryotic cell lines

Policy information about cell lines

| | |
|---|---|
| Cell line source(s) | Vero E6 and 293T cells were obtained from ATCC. FreeStyle293F cells were obtained from ThermoFisher |
| Authentication | Cell lines were checked for expression of ACE2 by FACS analysis. None of the cell lines were authenticated by karyotyping or other genomic techniques. |
| Mycoplasma contamination | Negative for Mycoplasma |
| Commonly misidentified lines (See ICLAC register) | No misidentified cell lines were used in the study. |

## Human research participants

Policy information about studies involving human research participants

| | |
|---|---|
| Population characteristics | Participants in this study were all adults aged 41-91 years old. Individuals of all ages and any gender that were hospitalized with COVID-19 disease were eligible for the study. |
| Recruitment | All adults hospitalized with COVID-19 disease in Maryland were eligible without any specific selection criteria. Samples were collected from patients who provided informed consent to participate in the study during the pandemic. |
| Ethics oversight | This study protocol was approved Food and Drug Administration's Research Involving Human Subjects Committee (RIHSC study protocol #2020-04-02). |

Note that full information on the approval of the study protocol must also be provided in the manuscript.
